# Global regulatory collaboration for the assessment of lenacapavir as a long-acting pre-exposure prophylaxis against HIV infection

**DOI:** 10.3389/fpubh.2026.1760130

**Published:** 2026-02-16

**Authors:** Hendy Kristyanto, María Isabel González-Tomé, Viktor Vlcek, Filip Josephson, Patrick Vrijlandt, Nicholas Perombelon

**Affiliations:** 1 European Medicines Agency, Amsterdam, Netherlands; 2 Swedish Medical Products Agency, Uppsala, Sweden; 3 Dutch Medicines Evaluation Board, Utrecht, Netherlands; 4 Department of Clinical Pharmacy and Pharmacology, University Medical Centre Groningen, Groningen, Netherlands

**Keywords:** EU medicines for all (EU-M4all), European Medicines Agency, HIV prevention, lenacapavir, long-acting PrEP, regulatory collaboration

## 1 Introduction

The availability of long-acting pre-exposure prophylaxis (PrEP) to prevent HIV infection will be essential for disease control. Despite significant improvements in prevention strategies, the global HIV population was estimated to increase by 1.3 million in 2024, with Africa accounting for nearly half of all new carriers (1). Although PrEP has been recommended since 2015 by the World Health Organization (WHO) for people at an increased risk of infection (2), uptake has been limited, and adherence is frequently inadequate. Daily oral PrEP is safe and effective in preventing HIV, particularly amongst men who have sex with men (MSM), serodiscordant couples, and people who inject drugs (PWIDs) (3). In addition, event-driven (on-demand) oral PrEP has been shown to be as effective as a daily dose amongst MSM in Europe (4). Notably, the efficacy was strongly associated with the levels of adherence. It is estimated that less than 30% of people who start using daily oral PrEP receive the HIV protective benefit of PrEP within 6 months of initiation due to discontinuation or poor adherence (5). Oral PrEP discontinuation rates are highest in sub-Saharan Africa, which has the greatest HIV-1 burden (5).

The first evidence of the benefits of long-acting PrEP came from the development program of cabotegravir. People taking an injection once every 2 months showed better adherence and a reduced risk of HIV infection compared with daily oral tenofovir disoproxil fumarate/emtricitabine (TDF/FTC) (6). Long-acting PrEPs with even lower dosing frequencies are expected to increase adherence and hence, effectiveness.

## 2 Global collaborative assessment of lenacapavir

Lenacapavir is a long-acting inhibitor of HIV-1 capsid function that is injected subcutaneously every 6 months. In 2022, it was authorized in the EU for the treatment of multidrug-resistant HIV-1 infection in combination with other antiretroviral therapies (7). On 24 July 2025, the Committee for Medicinal Products for Human Use (CHMP) of the European Medicines Agency (EMA) finalized its evaluation of lenacapavir for PrEP and considered the benefit–risk balance to be positive, recommending the marketing authorization of lenacapavir for PrEP, in combination with safer sex practices, to reduce the risk of sexually acquired HIV-1 infection in adults and adolescents with increased HIV-1 acquisition risk (8, 9).

Lenacapavir PrEP is the first medicine that was evaluated by EMA under accelerated assessment for a parallel EU-wide authorization [marketed as Yeytuo (8)] and the use outside the EU (9) via the EU medicines for all (EU-M4all) procedure. EU-M4all aims to facilitate patient access to essential medicines that prevent or treat diseases of major public health interest in low- and middle-income countries (LMICs) based on an EU scientific opinion (10). During the assessment of lenacapavir PrEP, experts from WHO and national regulatory authorities (NRAs) from Kenya, Nigeria, South Africa, Thailand, Uganda, Vietnam, Zambia, and Zimbabwe contributed to the scientific discussions by adding geographical considerations and perspectives on healthcare and pharmacovigilance systems of their countries. This inclusive approach supports regulatory harmonization and strengthens global capacity to protect public health. The accelerated assessment for lenacapavir shortened the timeline for review to 120 days instead of the standard 210 days since EMA considered that this product was of major public health interest.

Evidence of efficacy submitted in the application was demonstrated by the results of two phase three studies in people at an increased risk of infection: PURPOSE 1, involving 8,094 adolescent and adult cisgender women, and PURPOSE 2, involving 4,634 adolescent and adult men, and gender-diverse persons (11, 12). The primary analyses showed that HIV-1 incidence in the lenacapavir group was lower compared with an estimate of background HIV-1 incidence. However, CHMP considered the comparison unreliable, as it assumed that participants who were HIV-1 negative at screening and, therefore, were eligible for the randomized phase of the studies would subsequently contract HIV-1 at the same rate as the entire screened population, including those who were HIV-1 positive at screening and, consequently, were not eligible for the randomized phase. Secondary analyses, however, showed a clinically and statistically significant reduction in the incidence of HIV-1 infections with lenacapavir compared with daily oral TDF/FTC (PURPOSE 1: rate ratio (RR): 0.000, 95% confidence interval (CI): 0.000, 0.101; *p* < 0.0001; PURPOSE 2: RR: 0.111; 95% CI: 0.024, 0.513; *p* = 0.00245) (8, 9).

Importantly, treatment adherence was higher amongst participants who received lenacapavir compared with those given daily oral TDF/FTC. At week 52, 93.5% (PURPOSE 1) and 92.7% (PURPOSE 2) participants in the lenacapavir group received their injections on time, whilst fewer than 62% of participants in the TDF/FTC group showed high levels of tenofovir diphosphate in dried-blood-spot samples (11, 12).

Safety data for up to 12 months of exposure from 4,323 participants were considered adequate for assessment. Overall, lenacapavir was well-tolerated at the authorized dose and method of administration. The most common adverse reactions were mild to moderate injection site reactions (68.8–83.2%), which included slow or non-resolving injection site nodules (35.5–35.9%) (8, 9). EMA will review new safety data regularly (7–9).

The available data on the use of lenacapavir PrEP in pregnant and breastfeeding people show that the medicine may be considered in these groups if the expected benefit outweighs the potential risk to the fetus or child (8, 9). A registry study on antiretroviral (ARV) use during pregnancy is ongoing to generate additional data on its use in pregnancy and breastfeeding.

For transgender individuals, co-administration of lenacapavir with gender-affirming hormones estradiol and testosterone did not lead to clinically relevant interactions. Plasma concentration of progesterone may be increased when co-administered with lenacapavir. Interaction with anti-androgens was not studied. Based on the available data, no dose adjustment of these gender-affirming hormones is recommended when co-administered with lenacapavir (8, 9).

Finally, there is a risk of developing resistance to lenacapavir if HIV-1 infection occurs before initiation, during treatment, or after discontinuation of the medicine. To mitigate this risk, HIV-1-negative status must be confirmed prior to starting lenacapavir, before each subsequent injection, and monitored as clinically indicated. A combined antigen/antibody test and an HIV-RNA test should yield negative results. If combined testing is not available, testing should follow local guidelines. Individuals who are confirmed to have HIV-1 should immediately begin a complete ARV regimen to reduce the likelihood of resistance development. Lenacapavir-resistant HIV-1 variants retain susceptibility to all first-line ARV classes owing to lenacapavir's novel mechanism of action. Therefore, the risk is considered to have minimal clinical impact. This risk will be further characterized in a non-interventional study (8, 9).

## 3 Discussion

Lenacapavir provides a valuable additional long-acting option in the HIV PrEP armamentarium and brings clinical benefits in diverse at-risk populations based on high efficacy and a long administration interval, which prompts improved adherence compared to oral PrEP. The WHO has recommended lenacapavir as an additional HIV prevention modality to be integrated into combination HIV prevention approaches (13). Provided wide access, long-acting PrEPs are expected to have a global impact by improving HIV PrEP uptake and adherence. The scientific opinion from the CHMP will facilitate the WHO prequalification and registration of lenacapavir in LMICs. Following WHO prequalification, the NRAs in LMICs can make their own regulatory decisions, ensuring regulatory autonomy and local contexts. Accordingly, the South African Health Products Regulatory Authority (SAHPRA), the Zambia Medicines Regulatory Authority (ZAMRA), and Zimbabwe's Medicines Control Authority (MCAZ) have recently registered lenacapavir for PrEP in record time by adopting the CHMP scientific opinion as the result of the EU-M4All procedure. Other NRAs that were involved in the EU-M4All procedure are expected to follow suit. The registration of long-acting PrEPs is a prerequisite for the marketing authorization application of their generic products. This procedure exemplifies the need for regulatory collaboration to facilitate timely access to medicinal products addressing global health needs. Moreover, pricing agreements, early implementation studies, and expansion of access to lenacapavir in LMICs have been announced (14–16). These global initiatives and collaborations will foster early and equitable access to long-acting HIV PrEPs, particularly in countries with a high burden of HIV-1.

## References

[1] The World Health Organization. HIV Statistics, Globally and by WHO Region (2025). Available online at: https://cdn.who.int/media/docs/default-source/hq-hiv-hepatitis-and-stis-library/who-ias-hiv-statistics_2025-new.pdf?sfvrsn=5023deae_15 (Accessed August 19, 2025).

[2] The World Health Organization. Pre-exposure Prophylaxis (PrEP). Available online at: https://www.who.int/teams/global-hiv-hepatitis-and-stis-programmes/hiv/prevention/pre-exposure-prophylaxis (Accessed August 26, 2025).

[3] Murchu E Marshall L Teljeur C Harrington P Hayes C Moran P . Oral pre-exposure prophylaxis (PrEP) to prevent HIV: a systematic review and meta-analysis of clinical effectiveness, safety, adherence and risk compensation in all populations. BMJ Open. (2022) 12:e048478 doi: 10.1136/bmjopen-2020-048478 35545381 PMC9096492

[4] Molina J-M Ghosn J Assoumou L Delaugerre C Algarte-Genin M Pialoux G . Daily and on-demand HIV pre-exposure prophylaxis with emtricitabine and tenofovir disoproxil (ANRS PREVENIR): a prospective observational cohort study. Lancet HIV. (2022) 9:e554–62. doi: 10.1016/S2352-3018(22)00133-3 35772417

[5] Zhang J Li C Xu J Hu Z Rutstein SE Tucker JD . Discontinuation, suboptimal adherence, and reinitiation of oral HIV pre-exposure prophylaxis: a global systematic review and meta-analysis. Lancet HIV. (2022) 9:e254–68. doi: 10.1016/S2352-3018(22)00030-3 35364026 PMC9124596

[6] Chou R Spencer H Bougatsos C Blazina I Ahmed A Selph S. Preexposure prophylaxis for the prevention of HIV: updated evidence report and systematic review for the US Preventive Services Task Force. JAMA. (2023) 330:746–63. doi: 10.1001/jama.2023.9865 37606667

[7] The European Medicines Agency. Sunlenca (2025). Available online at: https://www.ema.europa.eu/en/medicines/human/EPAR/sunlenca (Accessed August 19, 2025).

[8] The European Medicines Agency. Yeytuo (2025). Available online at: https://www.ema.europa.eu/en/medicines/human/EPAR/yeytuo (Accessed August 19, 2025).

[9] The European Medicines Agency. Lenacapavir Gilead (2025). Available online at: https://www.ema.europa.eu/en/opinion-medicine-use-outside-EU/human/lenacapavir-gilead (Accessed August 19, 2025).

[10] The European Medicines Agency. Medicines for Use Outside the European Union. Available online at: https://www.ema.europa.eu/en/human-regulatory-overview/marketing-authorisation/medicines-use-outside-european-union (Accessed August 27, 2025).

[11] Bekker LG Das M Abdool Karim Q Ahmed K Batting J Brumskine W . Twice-yearly lenacapavir or daily F/TAF for HIV prevention in cisgender women. N Engl J Med. (2024) 391:1179–92. doi: 10.1056/NEJMoa2407001 39046157

[12] Kelley CF Acevedo-Quiñones M Agwu AL Avihingsanon A Benson P Blumenthal J . Twice-yearly lenacapavir for HIV prevention in men and gender-diverse persons. N Engl J Med. (2025) 392:1261–76. doi: 10.1056/NEJMoa2411858 39602624

[13] The World Health Organization. Guidelines on Lenacapavir for HIV Prevention and Testing Strategies for Long-Acting Injectable Preexposure Prophylaxis (PrEP) (2025). Geneva: World Health Organization. 40743386

[14] Unitaid. Unitaid, CHAI, and Wits RHI Enter into a Landmark Agreement with Dr. Reddy's to Make HIV Prevention Tool Lenacapavir Affordable in LMICs (2025). Available online at: https://unitaid.org/news-blog/lenacapavir-for-hiv-prevention/ (Accessed January 15, 2026).

[15] Unitaid. Lenacapavir Use Kicks off in South Africa and Brazil Through Unitaid Partnership with Wits RHI and Fiocruz (2025). Available online at: https://unitaid.org/news-blog/lenacapavir-use-kicks-off-in-south-africa-and-brazil-through-unitaid-partnership-with-wits-rhi-and-fiocruz/ (Accessed January 16, 2026).

[16] Unitaid. Unitaid Approves New Investments to Accelerate Equitable Access to Lenacapavir for HIV Prevention (2026). Available online at: https://unitaid.org/news-blog/unitaid-approves-new-investments-to-accelerate-equitable-access-to-lenacapavir-for-hiv-prevention/ (Accessed January 16, 2026).

